# Single Eye mRNA-Seq Reveals Normalisation of the Retinal Microglial Transcriptome Following Acute Inflammation

**DOI:** 10.3389/fimmu.2019.03033

**Published:** 2020-01-09

**Authors:** Oliver H. Bell, David A. Copland, Amy Ward, Lindsay B. Nicholson, Clemens A. K. Lange, Colin J. Chu, Andrew D. Dick

**Affiliations:** ^1^Academic Unit of Ophthalmology, Translational Health Sciences, University of Bristol, Bristol, United Kingdom; ^2^Eye Clinic, Medical Centre, University of Freiburg, Freiburg, Germany; ^3^Faculty of Medicine, University of Freiburg, Freiburg, Germany; ^4^Institute of Ophthalmology and the National Institute for Health Research Biomedical Research Centre, Moorfields Eye Hospital and University College London, London, United Kingdom

**Keywords:** microglia, transcriptome, uveitis, retina, resolution, mRNA-Seq, lipopolysaccharide (LPS), heterogeneity

## Abstract

**Background:** Whether retinal microglia can maintain or restore immune homeostasis during and after inflammation is unclear. We performed single-eye mRNA-sequencing on microglia at different timepoints following a single inflammatory stimulus to characterise their transcriptome during and after resolution of endotoxin-induced uveitis (EIU).

**Experimental Approach:** *Cx3cr1*^*CreER*^*:R26-tdTomato* (C57BL/6) male heterozygotes were administered tamoxifen via different regimes at 4–5 weeks of age. Four weeks post-tamoxifen, mice were injected intravitreally with 10 ng lipopolysaccharide (endotoxin induced uveitis, EIU). Six-hundred retinal microglia were obtained by FACS from individual naïve retinas and at 4 h, 18 h, and 2 weeks following EIU induction. Samples were sequenced to a depth of up to 16.7 million reads using the SMART-Seq v4 Ultra Low Input RNA kit. The data was analysed using Partek software and Ingenuity Pathway Analysis. Genes were considered differentially-expressed (DEG) if the FDR step-up *p*-value was ≤0.05 and the fold-change was ≥±2.

**Results:** Flow cytometric analysis indicates that the *Cx3cr1*^*CreER*^*:R26-tdTomato* strain is both sensitive (>95% tagging) and specific (>95% specificity) for microglia when tamoxifen is administered topically to the eye for 3 days. During “early” activation, 613 DEGs were identified. In contrast, 537 DEGs were observed during peak cellular infiltrate and none at 2 weeks, compared to baseline controls (1,069 total unique DEGs). Key marker changes were validated by qPCR, flow cytometry, and fluorescence microscopy. C5AR1 was identified and validated as a robust marker of differentiating microglial subsets during an LPS response.

**Conclusion:** Using EIU to provide a single defined inflammatory stimulus, mRNA-Seq identified acute transcriptional changes in retinal microglia which returned to their original transcriptome after 2 weeks. Yolk-sac derived microglia are capable of restoring their homeostatic state after acute inflammation.

## Introduction

It has been challenging to investigate microglia during inflammatory contexts primarily because distinguishing them from infiltrating monocytes and macrophages has been unreliable due to similar expression of cell-surface markers (1–6). Microglia contribute to an immunosuppressive environment, are derived from the yolk-sac, reside within two distinct niches in the retina, and show differential regulation because of inherent microglial heterogeneity (7, 8). Nonetheless conflicting data remains on how microglia regulate or promote inflammation depending on insult (9, 10). During inflammation microglia alter their homeostatic state (8, 11–13). In acute inflammation, markers including *C5ar1* show promise in delineating sub-populations of microglia mounting an LPS response (13). We wished to determine if the microglial transcriptome resets after an acute and resolving insult, or if homeostatic thresholds have been reset or altered permanently.

Recent advancements in transgenic mouse lines, but also in identification of markers that are “microglial-specific,” for example *Cd44, P2ry12, Siglech*, and *Tmem119*, have contributed in various degrees toward microglial identification (12, 14–20). However, loss of these markers at the transcript level are observed when microglia are perturbed and some of these markers are lost at the protein level, highlighting the need for careful validation of these markers as microglial-specific given their expression is dependent upon the disease context (8, 11–13). The *Cx3cr1*^*CreER*^*:R26-tdTomato* mouse strain permits binary discrimination of the microglia from other immune cells. The model utilises the high expression of *Cx3cr1* in microglia and the longevity (as low level self-replication) of microglia in comparison to other immune cells (14).

In this study, we validate the *Cx3cr1*^*CreER*^*:R26-tdTomato* strain as sensitive and specific for tagging retinal microglia and perform mRNA-Sequencing on microglia obtained from individual retina during and after resolution of acute inflammation induced by intravitreal injection of LPS [the endotoxin-induced uveitis (EIU) model]. We show that the retinal microglia undergo acute transcriptional changes which resolve to their original homeostatic state by 2 weeks and support microglial heterogeneity in response to inflammatory signals.

## Materials and Methods

### Mice

*Cx3cr1*^*CreER*^*:R26-tdTomato* mice on a C57BL/6J background were provided by Clemens Lange (University of Freiburg, Germany). Breeding colonies of homozygotes were established, and offspring crossed with C57BL/6J mice to generate heterozygotes for experiments. Genotyping (via PCR) of breeding pairs was performed. Mice were confirmed as negative for the *Rd8* mutation (21).

All mice were housed at the University of Bristol Animal Services Unit under specific pathogen free conditions with food and water *ad libitum*. All procedures were conducted in concordance with the United Kingdom Home Office licence (PPL 30/3281) and were approved by the University of Bristol Ethical Review Group. The study also complied with the Association for Research in Vision and Ophthalmology (ARVO) Statement for the Use of Animals in Ophthalmic and Visual Research.

#### Tamoxifen Preparation and Administration

Tamoxifen (T5648; Sigma-Aldrich, Poole, UK) was dissolved in corn oil (C8267; Sigma-Aldrich) to a concentration of 20 or 5 mg/mL, for subcutaneous injection and topical administration, respectively. The solutions were freshly prepared by overnight incubation in an orbital shaker at 42°C and 300 rpm. Mice were injected with 200 μL subcutaneously [100 μL into both the lower (inguinal) left and right quadrants using a 25G needle] on days 1 and 3; alternatively, mice were administered 10 μL topically to the eye three times daily (minimum gap of 2 h between dosing) for up to 4 days.

#### Induction of EIU

Prior to anesthesia, pupils were dilated using topical tropicamide 1% w/v and phenylephrine 2.5% w/v (Minims; Chauvin Pharmaceuticals, Romford, UK). Mice were anaesthetised by intraperitoneal injection of 90 μL/10 g body weight of a solution containing 6 mg/mL ketamine (Ketavet; Zoetis Ireland Ltd., Dublin, Ireland) and 2 mg/mL Xylazine (Rompun; Bayer plc, Newbury, UK) mixed with sterile water.

Mice were selected for injection (or to be used as a control) in a constrained randomised order within blocks using Excel 2016 (Microsoft, Redmond, WA); blocks were dependent on cage allocations, which itself was dependent on the litter they were derived from. The allocations ensured that all experiments had littermate controls.

Intravitreal injections were performed as previously described (22). In brief, 2 μL volume of PBS containing 10 ng LPS from *E. coli* 055:B5 (Sigma-Aldrich) was delivered into the intravitreal space via the pars plana, using an operating microscope and a 33-gauge needle on a microsyringe (Hamilton Company, Reno, NV) under direct visualisation. Immediately following injection, 1% w/w chloramphenicol ointment (Martindale Pharma, Romford, UK) was applied topically, with the animals monitored and kept on a heat-pad during recovery.

#### EIU Clinical Assessment

At selected time-points (4 h, 18 h, and 2 weeks) post-injection, pupils were dilated and mice anaesthetised for clinical assessment. The Micron IV retinal imaging microscope (Phoenix Research Laboratories, Pleasanton, CA) was used to capture optical coherence tomography (OCT) scans, and brightfield and fluorescence fundal images.

Prior to imaging, the Micron IV CCD and OCT were calibrated in accordance with the manufacturer's protocol. The gain was set to +3 dB and the FPS to 15, or +12 dB and 2 for brightfield and tdTomato fluorescence imaging, respectively. For tdTomato imaging, a 550/25 nm bandpass excitation and 590 nm longpass emission filter were used (Edmund Optics, Barrington, NJ).

For OCT, the parameters were defined according to the manufacturers protocol, and scans were taken 30 times in rapid succession and averaged. Full-length B-scans were taken horizontally and vertically with the optic disc centered. Images were stored in the TIFF file format.

### Isolation and Flow Cytometric Phenotyping of Retinal Immune Cells

Eyes were dissected in 100 μL ice-cold PBS with aqueous, vitreous, and retina extracted by a limbal incision, lens removal and transfer into a 1.5 mL microcentrifuge tube. The tissue was mechanically disrupted by rapping the tube along an Eppendorf rack 12 times before transfer into a 96-well 60 μm nylon mesh filter plate (Merck Millipore, UK). The plate was centrifuged at 400 *xg* for 5 min and the supernatant aspirated. The remaining cell pellet was resuspended in FACS buffer (PBS supplemented with 3% v/v Foetal calf serum and 0.9 mg/mL sodium azide) and transferred to a 96-well V-bottom plate for FACS staining.

Single cell suspensions from spleen and brain were generated by mechanical dissociation through a 70 μm cell strainer with a syringe plunger. Cells were centrifuged at 400 *xg* for 3 min, erythrocytes lysed using ammonium-chloride-potassium (ACK) buffer, and cells resuspended in FACS buffer. Cells were incubated with purified rat anti-mouse CD16/32 Fc block [Clone 2.4G2; Becton Dickson (BD) Biosciences, Oxford, UK] and 7-aminoactinomycin D (7AAD; Thermo Fisher Scientific, Waltham, MA) diluted in FACS buffer for 20 min at 4°C. Cells were then incubated with an antibody cocktail containing fluorochrome-conjugated monoclonal antibodies (see Supplementary Table 1) against various mouse cell-surface markers for 20 min at 4°C. All mAbs were titrated and tested using control tissues.

Cell suspensions were acquired using a 4-laser Fortessa X-20 flow cytometer (BD Cytometry Systems, Oxford, UK). Compensation was performed using OneComp eBeads (Thermo Fisher Scientific), Anti-Rat Ig, κ/Negative Control Compensation Particles Set (BD Biosciences), or AbC total antibody compensation bead kit (Thermo Fisher Scientific). To compensate tdTomato, cell suspensions prepared from *Cx3cr1*^*CreER*^*R26-tdTomato* homozygote brains were used. Fluorescence-minus-one (FMO) controls were used to assist in gating of the markers selected for validation. Following acquisition, analysis was performed using FlowJo software (Treestar, San Carlos, CA).

#### Fluorescence-Activated Cell Sorting

Retinal single cell suspensions were re-suspended in 200 μL FACS buffer containing DRAQ7 (DR77524; Biostatus, Shepshed, UK). Live microglial cells (tdTomato^hi^ DRAQ7^−^) were immediately sorted using a BD Influx Cell Sorter. Retinal samples were prepared in small batches in order to maintain cell viability and ensure high quality RNA.

Cells were sorted into 0.2 mL endonuclease-free tubes containing 0.05 μL RNase inhibitor, 0.95 μL lysis buffer, and a variable amount of nuclease-free water depending on the number of cells collected: $9.5-\left(\frac{x}{850}*3\right)$ μL, where *x* is the number of cells isolated, using components of the SMART-Seq v4 Ultra Low Input RNA Kit for Sequencing (Takara Bio USA, Inc., Mountain View, CA). Samples were sorted in a constrained randomised order in blocks; blocks were made as small as possible and consisted of a balance of every experimental group to ensure that time- or order-dependent (batch) effects of sorting were mitigated.

### mRNA-Sequencing

#### Sample and Library Preparation

Samples were prepared using the SMART-Seq v4 Ultra Low Input RNA Kit for Sequencing, according to the user manual, to convert the mRNA into cDNA and amplify by Long Distance (LD) PCR (16 cycles for 600 cells). cDNA was isolated using the Agencourt AMPure XP Kit (Beckman Coulter, Brea, CA) and quantified using the Agilent High Sensitivity DNA Kit on an Agilent 2100 Bioanalyser (Agilent Technologies, Santa Clara, CA). The library preparation was performed using the Nextera XT DNA Library Preparation Kit (Illumina Inc., San Diego, CA).

#### Sequencing and Analysis

Samples were sequenced to depths of up to 16.7 million single-end 75 nt length reads per sample using the Illumina NextSeq 500/550 High Output v2 kit (75 cycles) on an Illumina NextSeq 500 Sequencing System. Image analysis, base calling, and generation of sequence reads were produced using the NextSeq Control Software v2.0 (NCS) and Real-Time Analysis Software v2 (RTA). Data was converted to FASTQ files using the bcl2fastq2 v2.20 software (Illumina Inc.).

Sequencing data was initially quality-checked using FastQC^1^, before alignment and initial analysis. The data was processed through an analysis pipeline using the Partek Flow (Build version 6.0.17.0614; Partek Inc., St. Louis, MO) software with the following task nodes (non-default parameters are specified in brackets): Trim adapters (inputting Nextera XT Index Kit v2 adapter sequences^2^, Trim bases (From 3′ end, 1 base), Trim bases [from 3′ end with minimum quality score (Phred) of 30], Align reads using STAR (2.5.3a using mm10 as the reference index), Quantify to transcriptome (Partek E/M using mm10 – Ensembl Transcripts release 89 as the reference index) (Supplementary Figures 1, 2).

The Partek Flow data output was further analysed using Partek Genomics Suite (PGS) (Version 6.6, Build 6.16.0812). PGS normalises data using the reads per kilobase million (RPKM) approach and performs differential gene expression analysis using an ANOVA model; a gene is considered differentially-expressed (DEG) if it has an FDR step-up *p* ≤ 0.05 and a fold-change ≥±2. The data was subsequently analysed for enrichment of GO terms and the KEGG pathways; a pathway is considered significantly-enriched if the enrichment score is ≥3 (equivalent to a *p* ≤ 0.05). The fold-change and *p*-values of all genes were then imported into Ingenuity Pathway Analysis (IPA) version 01-13 (Qiagen Bioinformatics, Aarhus, Denmark) and analysed according to the manual. Pathways were considered significantly altered if they had a *p* ≤ 0.05 and a z-score (directionality score) ≥±2. PGS and IPA were both used to generate figures.

### Quantitative PCR

The remaining cDNA generated from the sorted cells was used for transcript-level validation. qPCR was performed using the TaqMan Universal Master Mix II, with UNG (4440038) and TaqMan gene expression probes (4331182) on a Quantstudio 3 Real-Time PCR system (A28137; all products from Thermo Fisher Scientific). Samples were run in technical duplicate, using 1 ng as the input amount, and analysed using the equation: 2^Cq(mean (control))−Cq(sample)^.

The probes used were: *Bst2* (mm1609165_g1), *C5ar1* (mm00500292_s1), *Cd44* (mm01277161_m1), *Fas* (mm01204974_m1), *Lair1* (mm00618113_m1), *Mertk* (mm00434920_m1), *Milr1* (mm01242703_m1), *P2ry12* (mm01950543_s1), *Siglech* (mm00618627_m1), *Slamf1* (mm00443317_m1).

### Immunohistochemistry

Eyes from euthanised mice were enucleated and fixed in 4% v/v PFA for 1 h before dissection. The anterior portion of the eye (cornea, iris, ciliary body, and lens) was carefully removed and an eyecup prepared. The eyecup tissue was blocked in 5% v/v normal goat serum (Vector Laboratories, CA, USA), 1% v/v BSA, and 3% v/v Triton x-100 (both Sigma Aldrich) in PBS for 4 h at room temperature with gentle shaking. Eyecups were then incubated at 4°C overnight with a rabbit anti-mouse anti-RFP mAb (600-401-379; Rockland Immunochemicals Inc., Limerick, PA) and for target validation experiments a Super Bright 600-conjugated anti-mouse CD44 mAb (Supplementary Table 1) was used in combination. After thorough washing with PBS, samples were incubated overnight with the secondary antibody goat anti-rabbit Alexa-633 (A21070; Thermo Fisher Scientific). The eyecups were washed again, and the retina carefully removed and flatmounted in Vectashield hard-set antifade mounting media (H-1400; Vector Laboratories Ltd., Peterborough, UK) and imaged on a Leica SP5-AOBS confocal laser scanning microscope (Leica Microsystems Ltd., Wetzlar, Germany). Images were acquired with an xy pixel size ≤ 200 nm, and a z-step size of ≤ 400 nm.

### Statistical Analysis and Image Processing

Data were analysed using GraphPad Prism 7 software (GraphPad Software Inc., San Diego, CA). The One-way ANOVA with Tukey's multiple comparisons test was used to compare multiple groups of data to a control group. A *p* ≤ 0.05 was considered significant.

Huygens professional software (Scientific Volume Imaging B.V., Hilversum, The Netherlands) was used to deconvolve the Micron IV fluorescent images and fluorescence microscopy (Supplementary Figure 3). For the Micron images, the following parameters were used: lens immersion = 1.343 [refractive index of the 0.2% w/w carbomer eye gel (23)], embedding = 1.377 (24), peak emission = 581 nm, numerical aperture = 1.25, and xy pixel size of 130 nm; the background was estimated at 2 and a signal-to-noise ratio of 15 was used. Hot pixel correction (with a sensitivity of 4) was used prior to deconvolution. For fluorescence microscopy, the parameters were imported from the microscope and the default settings were used.

Microscopy images were processed using the Leica LAS X software (Leica Microsystems Ltd.) and FIJI [a distribution of ImageJ (25)]. Other images, and figures, were processed using Photoshop (Adobe Inc., San Jose, CA).

## Results

### Subcutaneous and Topical Tamoxifen Administration Regimes Tag Retinal Microglia in the *Cx3cr1^*CreER*^:R26-tdTomato* Mouse Line

Activation of the CreER system requires administration of tamoxifen or 4-hydroxytamoxifen, and various methods of administration are frequently employed such as subcutaneous injection and oral gavage (14, 26). Moreover, an efficacious 4-day topical regime for activating CreER systems within the eye was recently described (27). Therefore, we initially tested which tamoxifen administration regime tagged retinal microglia efficiently while minimising systemic immune cell labelling to preclude analysing cells subsequently recruited during inflammation.

**Figure 1:**
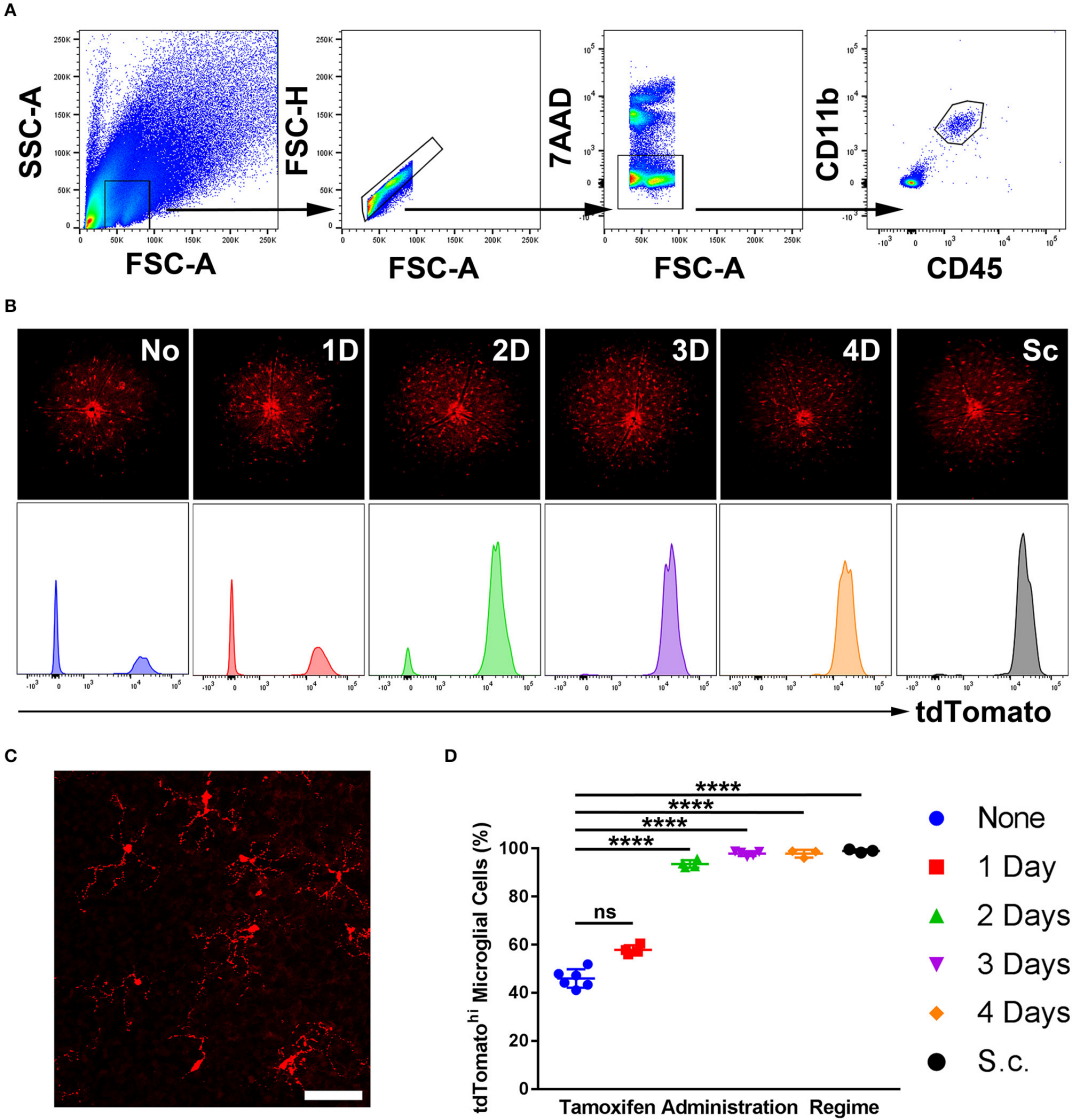
Sensitivity of microglial recombination induced in *Cx3cr1*^*CreER*^*:R26-tdTomato* mice using different tamoxifen administration regimes. **(A)** A representative flow cytometric gating strategy used to identify microglia in a naïve retina based on CD45 and CD11b expression. **(B)** Representative deconvolved tdTomato fluorescent fundal images of mice with representative tdTomato histograms (unit area scaling) on gated microglia from various tamoxifen administration regimes. **(C)** Confocal microscopy from a 3-day topical regime naïve retina shows microglia with a physiological ramified morphology, suggesting no gross perturbations in the microglia as a consequence of the transgenic model. **(D)** Aggregate data demonstrating the percentage of microglia that were tdTomato^hi^ (as quantified by flow cytometry) shows that a 3- and 4-day topical, in addition to subcutaneous, regimes result in full microglial tagging (*n* = 3–6). None, no tamoxifen administered; 1D, 1-day topical tamoxifen regime; 2D, 2-days topical tamoxifen regime; 3D, 3-days topical tamoxifen regime; 4D, 4-days topical tamoxifen regime; Sc, subcutaneous tamoxifen regime. ^****^p ≤ 0.0001, ns, not significant. Scale bar = 30 μm.

Five regimes were tested, spanning from 1- to 4-day topical and subcutaneous administration. A no-tamoxifen control was included. At 4 weeks post-tamoxifen treatment, flow cytometric analysis of the retinas quantified the proportion of CD45^int^ CD11b^+^ microglia (in a naïve retina) that were tdTomato^hi^ as corroborated with fluorescent fundal imaging (Figures 1A,B). Microglia displayed a “resting” ramified morphology (Figure 1C). The 3- and 4-day topical, and the subcutaneous regime tagged ≥95% of microglia whereas the no-tamoxifen controls had 45% tagged constitutively (Figure 1D). All tdTomato^hi^ cells were microglia, indicating specificity in the normal adult mouse retina.

### *Cx3cr1^*CreER*^:R26-tdTomato* Mice Exhibit Typical Kinetics of the EIU Model

**Figure 2:**
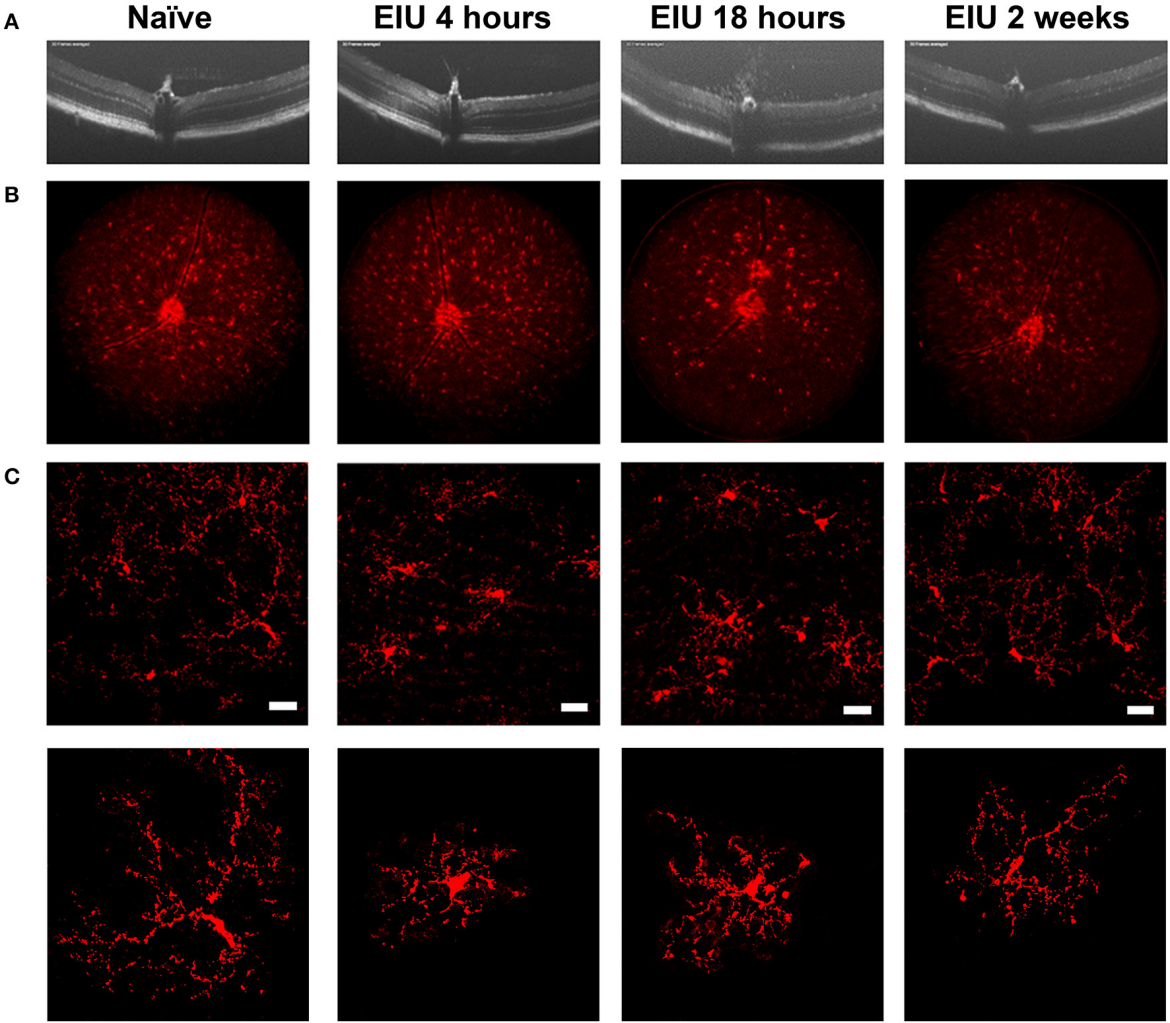
The kinetics of endotoxin-induced uveitis (EIU) in the *Cx3cr1*^*CreER*^*:R26-tdTomato* mouse strain. **(A)** OCT images showing disease-course in a single mouse (3-day topical regime), demonstrates the presence of infiltrating cells at 18 h post-injection with resolution by 2 weeks. **(B)** Deconvolved fluorescent fundal images acquired simultaneously show few changes in the distribution of tdTomato^+^ cells at 4 h post-injection, but changes at 18 h which resolve by 2 weeks. **(C)** Confocal microscopy of the tdTomato^+^ cells demonstrate a ramified toward amoeboid shift at 4- and 18 h post-injection that reverts to a ramified morphology by 2 weeks. Representative images of single cells are shown in the lower panel to highlight morphological changes. EIU, endotoxin-induced uveitis; OCT, optical coherence tomography. Scale bars = 30 μm.

The stability of microglial tagging during inflammation is pivotal to the isolation of pure populations of microglia for downstream transcriptomic assessment before, during, and after inflammation. To determine this, we utilised endotoxin-induced uveitis (EIU), a self-resolving model of acute Toll-like receptor 4 (TLR4)-mediated ocular inflammation, that following a single inflammatory insult generates acute immune cell tissue infiltration (28, 29). We confirmed disease kinetics in the *Cx3cr1*^*CreER*^*:R26-tdTomato* heterozygotes replicated our previous data in C57BL/6J mice (22). A time-course using OCT, deconvolved fluorescent fundal imaging, and confocal microscopy confirmed tdTomato expression throughout the expected time course of disease (Figure 2). Intravitreal administration of LPS results in peak cellular infiltrate at 18 h, which then resolves by 2 weeks (Figure 2A). Fluorescent fundal imaging demonstrates that the uniform distribution of microglia is altered in response to LPS, with defined areas of tdTomato^+^ cell accumulation observed at peak disease (Figure 2B). Confocal microscopy confirmed tdTomato^+^ cells in the naïve retina with a ramified morphology that became amoeboid at peak and recovered to a ramified appearance by 2 weeks post-EIU induction (Figure 2C).

### Three-Day Topical Tamoxifen Induction Ensures Specificity of tdTomato Labelling in Microglia

With tdTomato expression confirmed as stable in the microglial population, we sought to determine whether this was exclusive to microglia in the context of immune cell infiltration, validating accurate isolation of the microglial transcriptome. To confirm the specificity for retinal microglial tagging during inflammation, we examined tamoxifen regimes which efficiently tagged microglia (3- and 4-day topical, and subcutaneous) in addition to a no-tamoxifen control. Flow cytometry was performed on peripheral tissues and a small portion of myeloid cells were observed as tdTomato^hi^, primarily when tamoxifen was administered via the subcutaneous route (Supplementary Figure 4). However, to test the specificity within the retina, inflammation was required.

**Figure 3:**
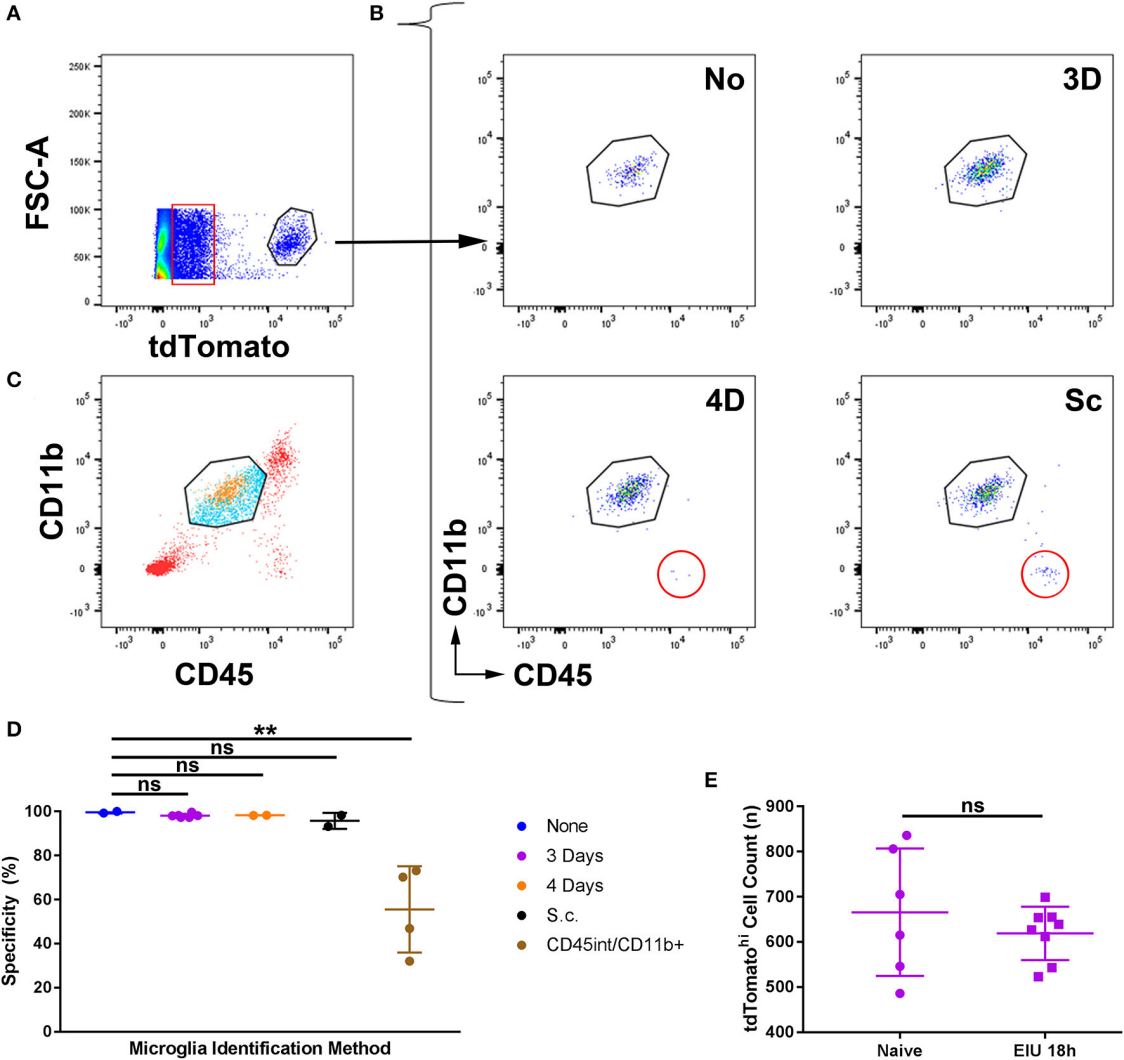
The specificity of microglial tagging for different tamoxifen administration regimes in the *Cx3cr1*^*CreER*^*:R26-tdTomato* mouse strain during active inflammation. **(A)** Peak EIU retinas following different tamoxifen administration regimes were prepared for flow cytometry. Live, cell singlets were gated based on tdTomato^hi^ expression. A small proportion (5%) of all live cells expressed low levels of tdTomato (boxed). **(B)** TdTomato^hi^ cells were gated based on CD45^int^ and CD11b^+^ expression, and in the 4-day and subcutaneous tamoxifen treated groups, non-microglial cells (CD45^hi^ CD11b^−^) were present (circled). **(C)** Gating microglia based on CD45 and CD11b expression alone results in the inclusion of infiltrating immune cells (tdTomato^−^, blue) in addition to retinal microglia (tdTomato^hi^, orange) but exclusion of cells not fitting the microglial expression profile (CD45^hi^/^lo^, red). **(D)** Aggregate data on the percentage specificity for microglia demonstrates that the 3-day topical route results in high specificity for microglia using tdTomato. The CD45^int^/CD11b^+^ group uses the microglial gating strategy (from live cell singlets without using tdTomato) shown in panel B for a comparison of the mouse strain to conventional microglial identification strategies (*n* = 2–5). **(E)** Total counts from naïve and peak EIU retinas, following the 3-day topical tamoxifen regime, demonstrate equivalent numbers of tdTomato^hi^ cells (*n* = 6–8). None, no tamoxifen administered; 3D, 3-days topical tamoxifen regime; 4D, 4-days topical tamoxifen regime; Sc, subcutaneous tamoxifen regime. ^**^p ≤0.01, ns, not significant.

Retinas were retrieved at the peak cellular infiltrate stage of EIU (18 h) and flow cytometry was performed (Supplementary Figure 5). Single, live cells identified as tdTomato^hi^ were assessed by conventional microglia markers of CD45^int^ and CD11b^+^ (Figures 3A,B). A small proportion (5%) of all single live retinal cells express very low levels of tdTomato (Figure 3A, boxed), as described in the tdTomato reporter mice (in the absence of Cre/Cre^ER^)^3^ due to a failure of a small proportion of ribosomes to terminate translation when reaching the stop codon that precedes *tdTomato* (30). Using this gating strategy, analysis from the 4-day topical and subcutaneous regimes demonstrates the presence of an additional population of non-microglial (CD45^hi^ CD11b^−^) cells that were identified in the tdTomato^hi^ subset (Figure 3B). Gating with CD45^int^ and CD11b^+^ but not tdTomato leads to the inclusion of non-microglial cells (Figure 3C) and greatly compromises specificity. The 3-day topical and no-tamoxifen control retained the specificity of tdTomato for microglia (Figures 3B,D), but as shown previously a no-tamoxifen control does not fully label the microglial population (Figure 1). As our approach for distinguishing microglia (CD45^int^ CD11b^+^ tdTomato^hi^) from the tdTomato^hi^ group could include non-microglia that possess a similar transcriptional profile to retinal microglia, the total counts of the CD45^int^ CD11b^+^ tdTomato^hi^ group, from naïve and peak EIU retinas (3-day topical tamoxifen), were compared and no significant difference was observed confirming a pure microglial population (Figure 3E). All subsequent experiments performed used the 3-day topical tamoxifen regime.

### Microglia Undergo Acute Transcriptional Changes During EIU That Fully Normalise to a Baseline State by 2 Weeks

The 3-day tamoxifen regime in the *Cx3cr1*^*CreER*^*:R26-tdTomato* strain provides the sensitivity and specificity required for reliable identification and isolation of distinct retinal microglia populations from other populations of infiltrating immune cells. To characterise changes in the microglial transcriptome during and after resolution of EIU, FACS (based on tdTomato) of 600 live microglia was performed from individual retinas collected at 4 h, 18 h, and 2 weeks post-injection in addition to naïve mice. The majority of these samples (28/30) yielded high-quality cDNA for sequencing using a validated pipeline (Supplementary Figure 1).

**Figure 4:**
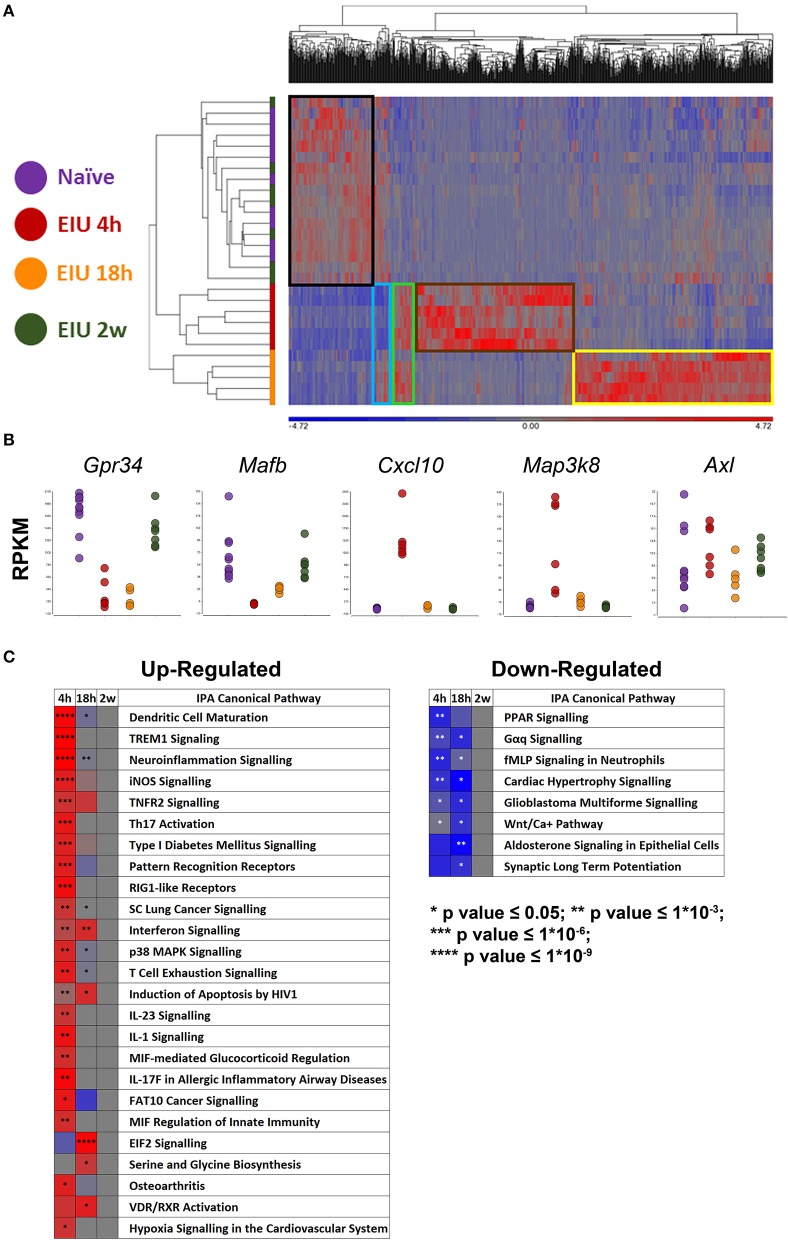
mRNA-Sequencing of microglia during and after EIU reveals transcriptional alterations that fully resolve. **(A)** Hierarchical clustering of differentially-expressed genes (DEGs) shows differences in the kinetics of the microglial transcriptome during EIU. Boxes highlight clusters of genes with different kinetics, and a restoration back to a homeostatic signal by 2 weeks (*n* = 5–10). **(B)** Scatterplots show changes in expression of previously described microglial genes to include a homeostatic gene (*Gpr34*), homeostatic transcription factor (*Mafb*), generic activation gene (*Cxcl10*), acute LPS-response gene (*Map3k8*), and “primed microglia” gene (*Axl*). **(C)** Heatmaps highlight canonical pathways, which were significantly different during at least one timepoint, change in direction (z-score) and *p*-value over time (*n* = 5–10); the pathways are in ascending order based on their overall (summary) *p*-value. The raw canonical pathway figures are presented in Supplementary Figure 6. EIU, endotoxin-induced uveitis. ^*^p ≤ 0.05, ^**^p ≤ 1*10^−3^, ^***^p ≤ 1*10^−6^, ^****^p ≤ 1*10^−9^.

Our approach identified 1,069 unique differentially expressed genes (DEGs; 613 at 4 h, 537 at 18 h, and 0 at 2 weeks) visualised by hierarchical clustering, revealing a highly plastic transcriptome with most up-regulated genes being mutually exclusive at different timepoints. Boxes highlight clusters of genes that were normal at 4 h but up-regulated at 18 h (yellow), those which were up-regulated at 4 h but not 18 h (brown), those which were up-regulated at both time-points (green), those which were down-regulated at 4 h but recovered to pre-EIU levels by 18 h (light blue), and those which were down-regulated at both 4- and 18 h (black). Restoration back to a homeostatic signal was observed by 2 weeks because unsupervised clustering failed to distinguish naïve and 2-week post-injection samples (Figure 4A). A multitude of expected gene changes based on published literature were observed, including downregulation of microglial homeostatic genes (*Gpr34, Mafb*), expression of microglial activation and LPS-response genes (*Cxcl10, Map3k8*), in addition to no change in the “primed microglia” gene *Axl* (11, 12, 17, 31) (Figure 4B). A heatmap demonstrates the change in z-score over time of canonical pathways identified as significantly different (Figure 4C). The original canonical pathways (including bars showing exact *p*-values) are presented in Supplementary Figure 6.

Enriched GO terms (enrichment score) when naïve and 4 h EIU groups were compared included: immune system process (60.9), regulation of cytokine production (56.0), and response to stress (52.7). Similarly, the enriched KEGG pathways included: NF-Kappa B signaling pathway (34.1), toll-like receptor signaling pathway (31.7), and TNF signaling pathway (25.3). For the naïve and 18 h EIU comparison GO terms included: cytosolic part (89.7), extracellular organelle (70.67), translation (65.8), and immune system process (29.8); enriched KEGG pathways included: ribosome (58.8) and proteasome (42.9). LPS was found to be a “master regulator” by IPA, indicating agreement of our data with the curated lists and pathways.

### Flow Cytometry and Fluorescence Microscopy Validate Key Transcriptional Alterations

**Figure 5:**
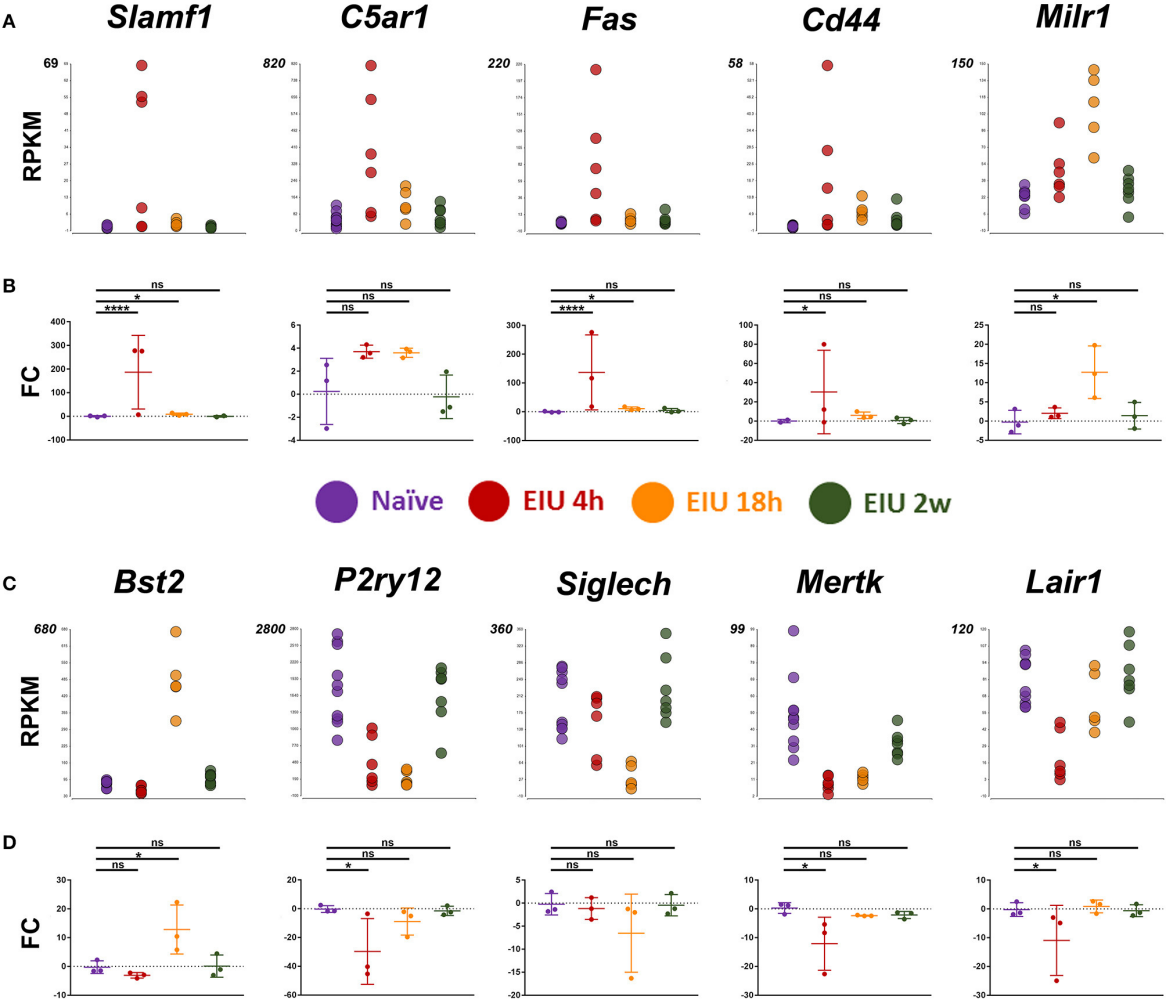
Transcript changes in selected markers, using cDNA generated from 600 sorted microglia, over a time-course of EIU. **(A,C)** RPKM values are shown for the 10 selected markers **(B,D)** in-line vertically with matching qPCR validation. EIU, endotoxin-induced uveitis; FC, Fold-change; RPKM, reads per kilobase of transcript per million mapped reads. ^*^p ≤ 0.05, ^****^p ≤ 0.0001, ns, not significant.

Our next aim was for orthogonal validation of key and novel transcriptional changes, both at the RNA and protein level. Markers for validation were selected by systematic assessment based on magnitude of the relative change in expression, novelty, lack of prior validation at the protein level, whether they were a previously suggested microglial marker, were in contrast to or appeared crucial in light of other reports, and lastly the availability of testing reagents (Supplementary Figure 7). Ultimately, the final set of 10 markers selected represent a variety of expression patterns and kinetics to match the plastic landscape identified by hierarchical clustering. In line with published reports and an activated state, pro-inflammatory markers (*Slamf1, C5ar1, Fas*, and *Cd44*) were all upregulated at 4 h following LPS challenge. In addition, a novel microglial associated transcript, *Milr1* (a negative regulator of mast cell activation) and *Bst2* (a previously validated marker of late activation) were elevated by 18 h. In contrast, constitutively expressed microglial genes, including homeostatic genes (e.g., *P2ry12, Siglech, Mertk*, and *Lair1*) were down-regulated at the early time-point. In general, qPCR analysis validated the transcript-level changes observed at each time-point, confirming resolution and return to baseline levels by 2 weeks (Figure 5).

**Figure 6:**
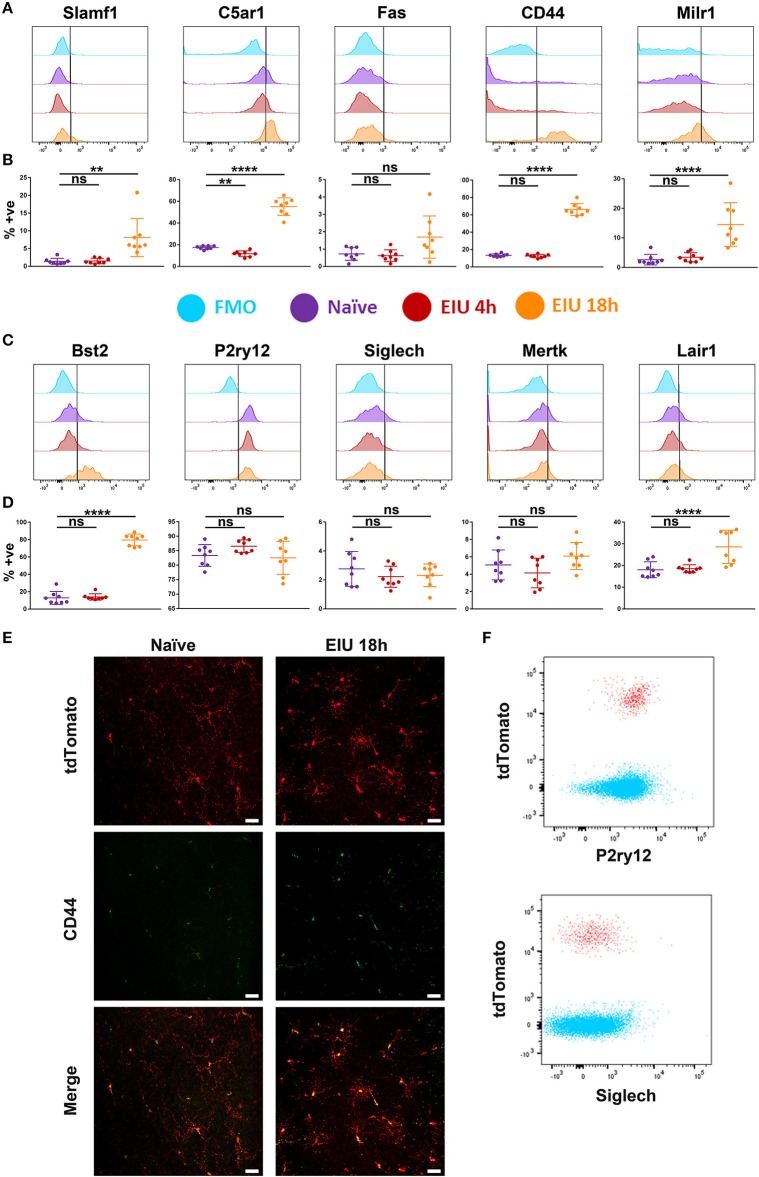
Changes in protein expression of selected markers in microglia over a time-course of EIU. **(A,C)** A representative flow cytometric histogram is shown for the 10 selected markers **(B,D)** in-line vertically with matching scatterplots of the aggregate flow cytometry data summarising the percentage of microglia positive for each marker at each timepoint. Gates were drawn with the assistance of fluorescence-minus-one (FMO) controls (light blue). **(E)** Confocal microscopy also confirms the upregulation of CD44 in microglia during EIU. **(F)** Flow cytometric analysis demonstrates P2RY12 and SIGLECH expression on CD45^+^ tdTomato^−^ non-microglial immune cells (blue) and CD45^+^ tdTomato^+^ microglia (red) at 18 h EIU. % +ve, percentage positive; EIU, endotoxin-induced uveitis. ^**^p ≤ 0.01, ^****^p ≤ 0.0001, ns, not significant. Scale bars = 30 μm.

Flow cytometric analysis demonstrates increased expression in SLAMF1, MILR1, C5AR1, CD44, BST2, and LAIR1 at 18 h post-injection (Figures 6A–D). Furthermore, differences in the proportion of marker-positive cells were evident, with C5AR1, CD44, and BST2 upregulated in the majority of microglia (>50%), in contrast to the other makers which were elevated in a smaller fraction (<20%) of cells. The upregulation of CD44 was also confirmed in retinal flatmounts at 18 h using fluorescence microscopy (Figure 6E). Whilst P2RY12 was highly expressed in naïve microglia (>80%), no change in expression in response to LPS were observed. Similarly, low level SIGLECH, MERTK, and FAS expression in naïve populations remained unchanged and restricted to a small percentage of the microglia (<10%). We also compared expression of P2RY12 and SIGLECH on CD45^+^ cells, as both are previously suggested markers that differentiate microglial populations from other immune cells (17, 20). Flow cytometric analysis clearly demonstrates that both markers are equally expressed on CD45^+^ infiltrating cells, indicating these markers exhibit poor specificity for retinal microglia during the acute response (Figure 6F).

### Stratifying Microglia Using C5AR1 Identifies Both Generalised and Restricted Microglial Responses

Recent reports show that *C5ar1* was one of several markers that was enriched in a subset of brain microglia (identified by sc-mRNA-Seq data) responding to systemic LPS challenge *in vivo* (13). Furthermore, mounting evidence from numerous reports identify heterogeneity in the microglial response during other pathological states (8, 32). We therefore examined whether stratifying microglia based on C5AR1 expression would delineate differences in the markers selected for validation, highlighting specificity to this subset of C5AR1-expressing cells, or generalised expression across the whole population of microglia.

**Figure 7:**
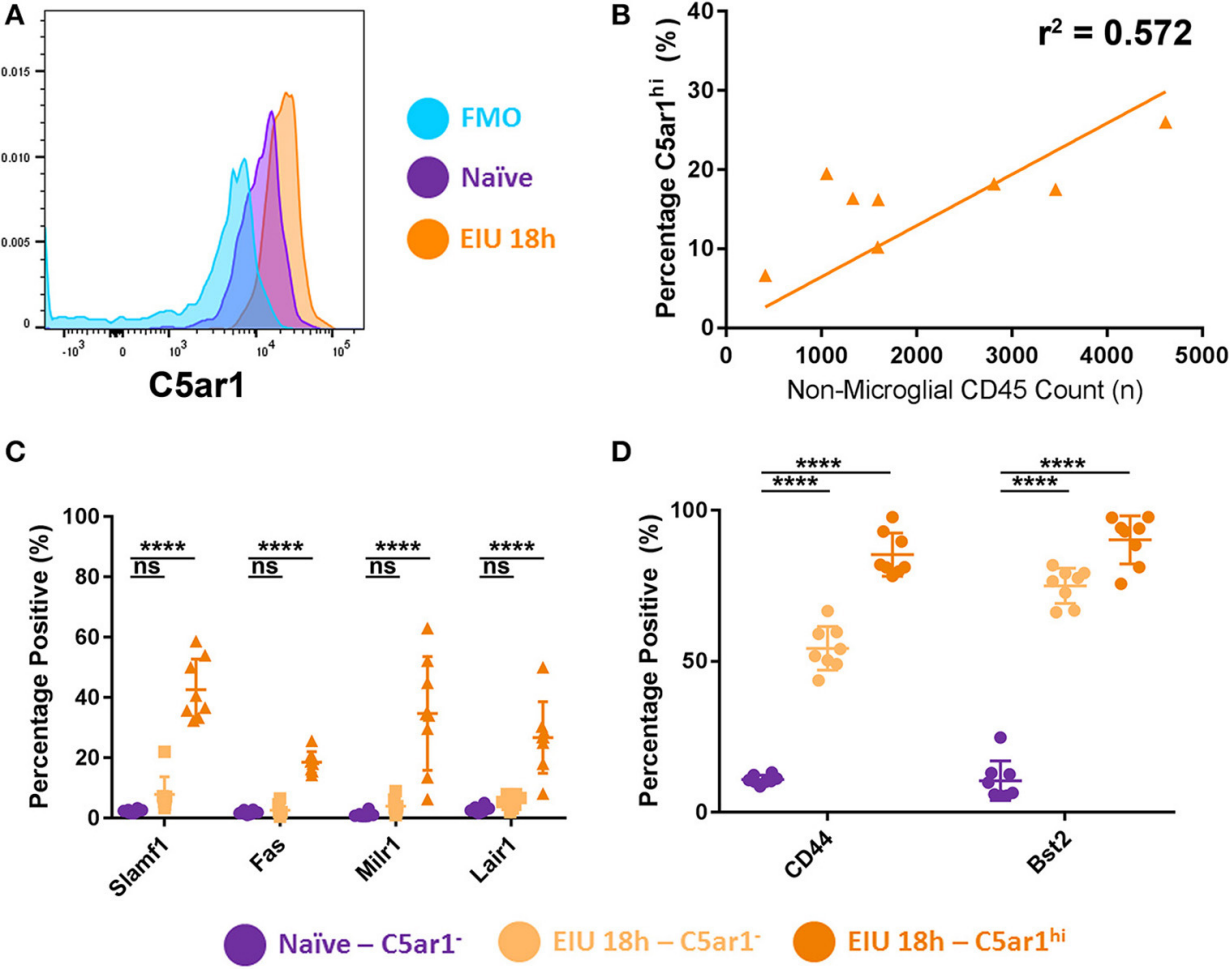
Stratifying microglia using C5AR1 expression identifies both restricted and generalised responses. **(A)** A histogram shows microglial C5AR1 expression in a fluorescence-minus-one (FMO) control (blue), naïve retina (purple), and at 18 h EIU (orange). **(B)** Immune cell infiltrate correlates with C5AR1^hi^ expression in microglial populations (*p* = 0.0298). **(C)** Stratifying microglia into C5AR1^−^ and C5AR1^hi^ (elevated above the naïve level of expression) identifies changes in cell-surface protein expression that are restricted to C5AR1-expressing microglia, **(D)** but also changes in proteins which are generalised microglial responses (not exclusive to, but somewhat enriched in, the C5AR1-expressing microglia). ^****^p ≤ 0.0001, ns, not significant.

Microglia were stratified into three main groups: C5AR1^neg^, C5AR1^lo^, and C5AR1^hi^. The C5AR1-expressing microglia were sub-stratified based on whether the C5AR1 expression was equivalent to microglia observed within a naïve mouse (C5AR1^lo^) or whether expression was elevated (C5AR1^hi^; Figure 7A). C5AR1^hi^ expression correlated to the extent of immune cell (CD45^+^ TdTomato^−^) infiltrate within the retinas and represents a potential microglial marker for disease scoring (Figure 7B). Flow cytometric analysis compared expression of the other surface markers in C5AR1^neg^ and C5AR1^hi^ subsets, as two distinctive populations, in naïve and peak EIU retinas. Delineating the two populations on this basis demonstrates elevation of several markers (SLAMF1, FAS, MILR1, and LAIR1) which are restricted to the C5AR1-expressing microglia (Figure 7C). In contrast CD44 and BST2 were enriched within the C5AR1-expressing population but also expressed by a large proportion of the C5AR1^neg^ microglia, thus representing more generalised markers of microglial perturbation (Figure 7D).

## Discussion

Single eye mRNA-Seq revealed inflammation-responsive transcriptional changes in retinal microglia following LPS stimulation which resolve within 2 weeks, confirming the potential for these cells to reset their homeostatic state. In line with the literature, our data confirms recognised patterns of altered gene expression in homeostatic and activation pathways, an absence of changes in “primed microglia” genes, and identifies enriched pathways relating to immune function.

Hierarchical clustering of the 1,069 DEGs identified reveals a high level of plasticity in the microglial transcriptome, with the majority of up-regulated genes at early (4 h) and peak (18 h) being mutually exclusive across the time-points. At the transcript level at least, this highlights a consensus group of microglial LPS-response genes including (*Slamf1, C5ar1, Fas, Cd44, Milr1*, and *Bst2*). In contrast, we highlight a common cluster of down-regulated genes, including homeostatic regulators (*P2ry12, Siglech, Mertk, Lair1*).

Orthogonal validation by qPCR of the 10 markers selected representing key and novel transcripts, confirmed the RNA-Seq findings. However, altered expression of some of these membrane-associated makers did not translate to changes at the protein level, as determined by flow cytometry. In part, this may reflect the presence of intracellular protein that our flow cytometric approach did not detect, or represents genuine discrepancies between the transcriptome and proteome, as these are not always in direct proportion (33–35). Our results emphasise caution in reading out RNA-Seq alone as a true representation of the cell's activity and highlight the need for orthogonal validation. Nonetheless, the single eye mRNA-Seq approach identified key and novel transcriptional changes which informed subsequent testing on a smaller number of markers via low-throughput approaches.

We confirmed and validated some of the transcriptional changes by flow cytometry but found that the differences were enhanced, and an additional marker (FAS) was identified as significantly different, when microglia were first stratified based on their C5AR1 expression as suggested via a transcripts-led report (13). Furthermore, we identified markers which were exclusive to the C5AR1-expressing microglia (SLAMF1, FAS, MILR1, and LAIR1), but also generalised markers which were not (CD44 and BST2). This agrees with other reports which suggest that C5AR1 is needed for microglial polarisation to pro-inflammatory states, and that its knock-out improved outcomes in an Alzheimer's model (36). Furthermore, microglial heterogeneity has already been reported in Alzheimer's disease, light-damage models, and in response to LPS stimulation *in vivo* (8, 13, 32). Understanding microglial heterogeneity, and identifying changes which are exclusive to subpopulations, is critical for developing targeted therapies.

Conflicting with previous reports investigating LPS-responses and experimental autoimmune encephalomyelitis (EAE), we did not find significant down-regulation of P2RY12 upon microglial activation (11, 13). In EAE, the microglia exhibit a chronic inflammatory state different to the acute LPS response, whilst the report investigating the LPS response used a systemic dose 400 times greater than our own local dose and had an endpoint 24 h post-injection which could explain the discrepancy. We suggest microglial loss of P2RY12 as context-dependent, for example when subject to a significant immune stimulus or persistent inflammation.

For this investigation, a clear marker to distinguish long-lived, yolk-sac derived microglia from infiltrating myeloid cells was critical, particularly during peak ocular inflammation at 18 h post-LPS injection. The *Cx3cr1*^*CreER*^*:R26-tdTomato* line has been validated for this approach previously (14, 37), but as we demonstrate the systemic tamoxifen administration also labels peripheral leukocyte populations which are recruited to the eye during inflammation. Modifying a recently published protocol (27) demonstrates that the 3-day topical tamoxifen administration regime is robust in labelling retinal microglia in both a sensitive (Figure 1) and specific (Figure 3) manner, superior to the established subcutaneous route. Shortening of the originally-published topical protocol from 4 days to three is likely possible due to the very high expression of *Cx3cr1* in microglia and reduces confounders of animal handling and stress to the mice as a refinement^4^ The high expression of *Cx3cr1* by microglia also explains why mice underwent tamoxifen-independent (or constitutive) recombination in 45% of the microglia as tamoxifen-independent recombination (constitutive receptor activation of the CreER) is a well-characterised phenomenon (38–40).

With other administration routes, we found that the specificity for microglia was reduced during ingress of immune cells with inflammation. This supports recent studies of microglia that highlight the need to confirm the specificity for microglia in their disease model and employ techniques with single-cell resolution to resolve the non-microglial cell populations (8, 41).

Taking all our data together, we show that three previously suggested markers (P2RY12, CD44, and SIGLECH) exhibit poor specificity for microglia. However, with the *Cx3cr1*^*CreER*^*:R26-tdTomato* line it remains possible to validate potential markers, assuming the line retains specificity for microglia across disease contexts. Microglial subtypes can exhibit both differential and generalised responses to LPS.

In summary, we demonstrate that the homeostatic threshold of retinal microglia is reset following an acute inflammatory insult and identify potential markers for delineating the heterogeneity of microglia that may be used depending on context of retinal perturbation.

## Data Availability Statement

The datasets generated for this study can be found in the Gene Expression Omnibus (GEO) repository under the accession number GSE138247. Spreadsheets of gene expression values, in addition to the lists of DEGs (with *p*-values and fold-changes) are included as part of this upload.

## Ethics Statement

All procedures were conducted in concordance with the United Kingdom Home Office licence (PPL 30/3281) and were approved by the University of Bristol Ethical Review Group. The study also complied with the Association for Research in Vision and Ophthalmology (ARVO) Statement for the Use of Animals in Ophthalmic and Visual Research.

## Author Contributions

OB, DC, AW, and CC performed the experiments. CL provided materials. OB, DC, CC, and AD planned and analysed the experiments. OB, DC, CC, LN, CL, and AD wrote the manuscript and planned the research project.

### Conflict of Interest

The authors declare that the research was conducted in the absence of any commercial or financial relationships that could be construed as a potential conflict of interest.

## Footnotes

^1^Simon Andrews, Brian Howard, Phil Ewels. FastQC: a quality control tool for high throughput sequence data 2015. Available online at: http://www.bioinformatics.babraham.ac.uk/projects/fastqc/.

^2^Illumina. Illumina Adapter Sequences 2017. Available online at: https://support.illumina.com/content/dam/illumina-support/documents/documentation/chemistry_documentation/experiment-design/illumina-adapter-sequences-1000000002694-11.pdf).

^3^The Jackson Laboratory. 007914 - B6.Cg-Gt(ROSA)26Sor 2019. Available online at: https://www.jax.org/strain/007914.

^4^National Centre for the Replacement R, Reduction of Animals in R. The 3Rs | NC3Rs 2018. Available online at: https://www.nc3rs.org.uk/the-3rs.
