# Supplementary material for: Single Eye mRNA-Seq Reveals Normalisation of the Retinal Microglial Transcriptome Following Acute Inflammation
Source: Front Immunol. 2020 Jan 9;10:3033. doi: 10.3389/fimmu.2019.03033 (PMC6964706; doi:10.3389/fimmu.2019.03033)
Supplement: Supplementary file 1 [file Data_Sheet_1.docx]

Supplementary Material

| Panel | Target | Fluorophore | Clone | Supplier |
| --- | --- | --- | --- | --- |
| Broad Immune Cell Phenotyping | B220 | FITC | RA3-6B2 | BD Biosciences |
|  | CD3 | APC | 145-2c11 |  |
| (Used in all panels) | CD45 | PE-Cy7 | 30-F11 |  |
|  | Gr-1 (Ly6C/Ly6G) | Alexa-700 | RB6-8C5 |  |
|  | CD11b | APC-Cy7 | M1/70 |  |
| Microglia Seq Validation | Milr1 (Allergin-1) | BV421 | TX83 |  |
|  | C5ar1 | BV510 | 20/70 |  |
|  | CD44 | Super Bright 600 | IM7 | Thermo Fisher Scientific |
|  | Siglech | BV650 | 440c | BD Biosciences |
|  | Fas (CD95) | BV711 | Jo2 |  |
|  | Mertk (Mer) | BV786 | 108928 |  |
|  | Slamf1 (CD150) | FITC | 9D1 | Thermo Fisher Scientific |
|  | Bst2 (CD317) | PE-eFluor610 | eBio927 |  |
|  | Lair1 | PE-Cy5.5 | 113 | Novus Biologicals* |
|  | P2ry12 | APC | S16007D | Biolegend |
| Supplementary Table 1. A list of monoclonal antibodies used for flow cytometry in the current study. The “Broad Immune Cell Phenotyping” panel was used for testing the sensitivity and specificity of microglial tagging, in addition to EIU kinetics. The “Microglia Seq Validation” panel was used to validate the novel and key makers identified by mRNA-Seq during EIU. *Novus Biologicals (Centennial, CO). | | | | |


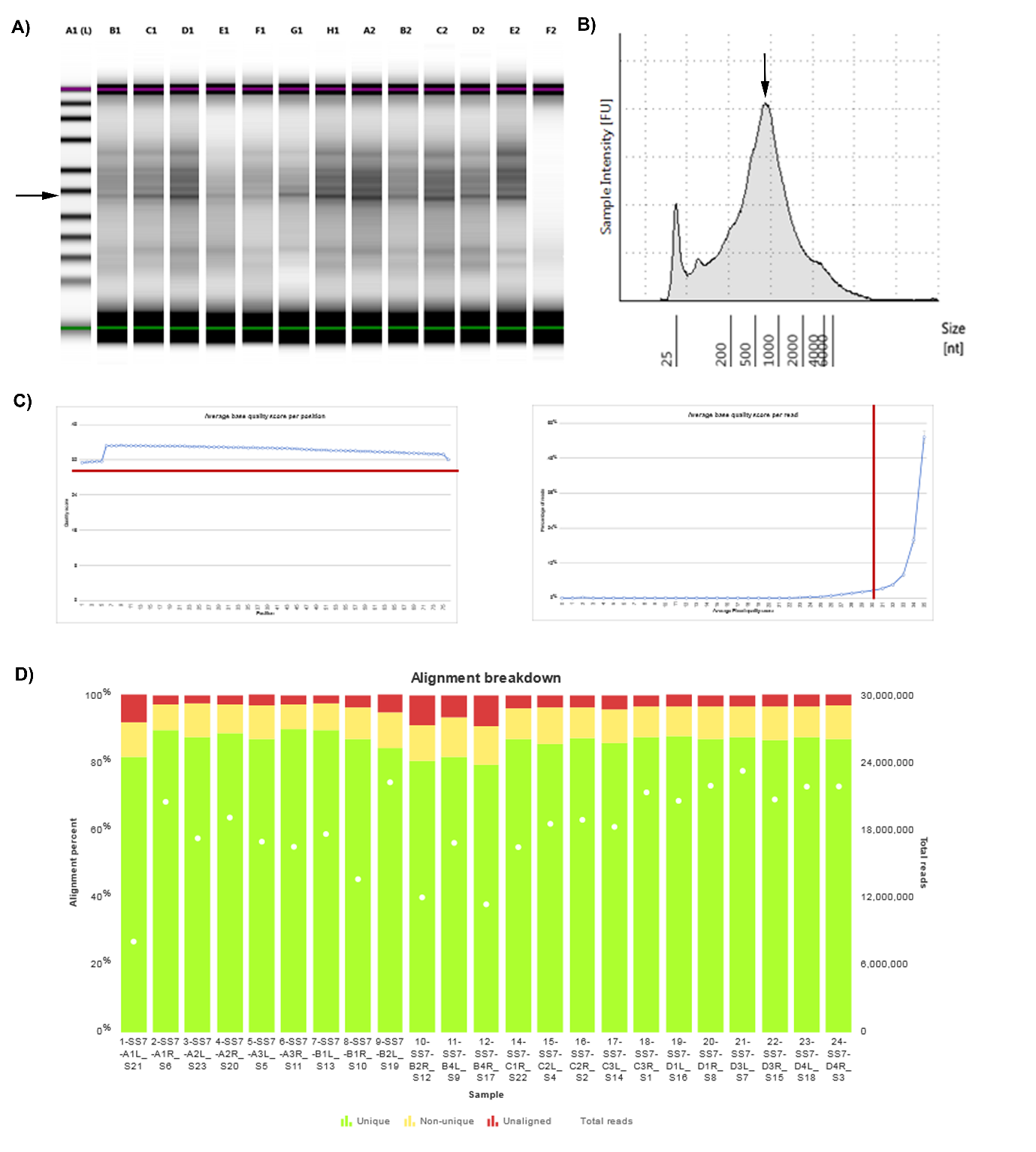


**Supplementary Figure 1. RNA-Seq QA/QC. (A)** A representative Agilent bioanalyser gel shows a distinct cDNA peak just below 1,000 bp (arrow) in samples, with no amplification in a negative water control in column F2. **(B)** A histogram of a single sample shows a single clean peak, indicating high quality output material. The FastQC report indicated a non-uniform distribution of bases that stabilised after the first 15 nucleotides (as expected), very low (if any) n content, and a normal GC content. **(C)** RNA-Seq QA/QC of Phred quality scores shows an average score >30 in all base positions (1-75; left), and a Phred score >30 for the vast majority of reads (right; ≤2.5% missed this criterion) – red lines indicate a Phred score of 30. **(D)** The percentage of total alignments (green and yellow) was ~90% per sample, with ~80% of all reads aligned uniquely (green).


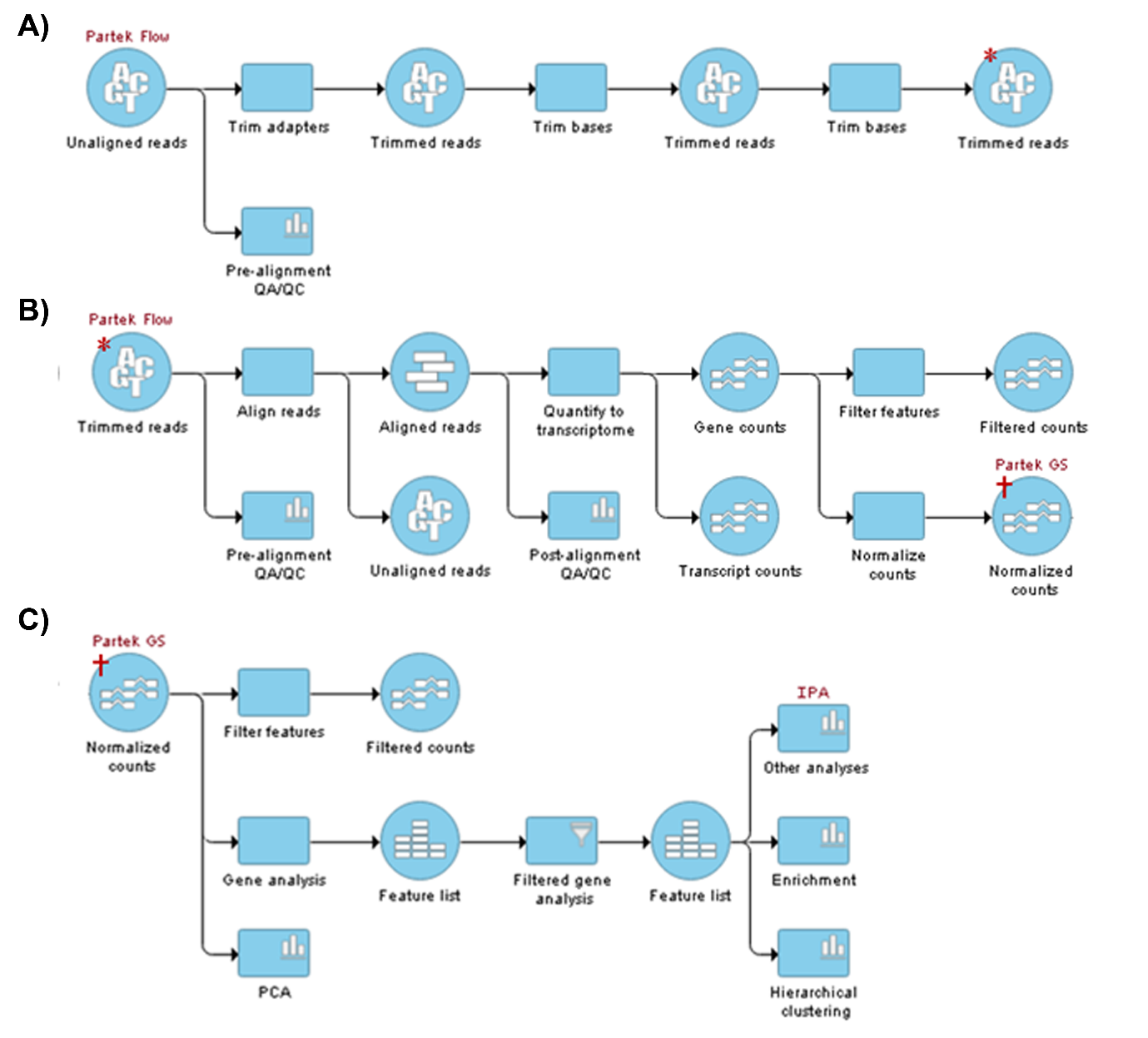


**Supplementary Figure 2. The bioinformatics pipeline for processing and analysing the RNA-Seq data. (A)** The bioinformatics analysis shown starting with the unaligned reads and pre-alignment processing, **(B)** alignment, alignment QC and quantification of reads, and **(C)** differential gene expression analysis (DGEA) and filtering/visualisation; Partek Flow was used for pre-processing, alignment, and quantification of the data whilst Partek Genomics Suite (PGS) was used to normalise the data and perform DGEA. Both PGS and Ingenuity Pathway Analysis (IPA) were used for visualisations and further analysis of the data and pathway enrichment.


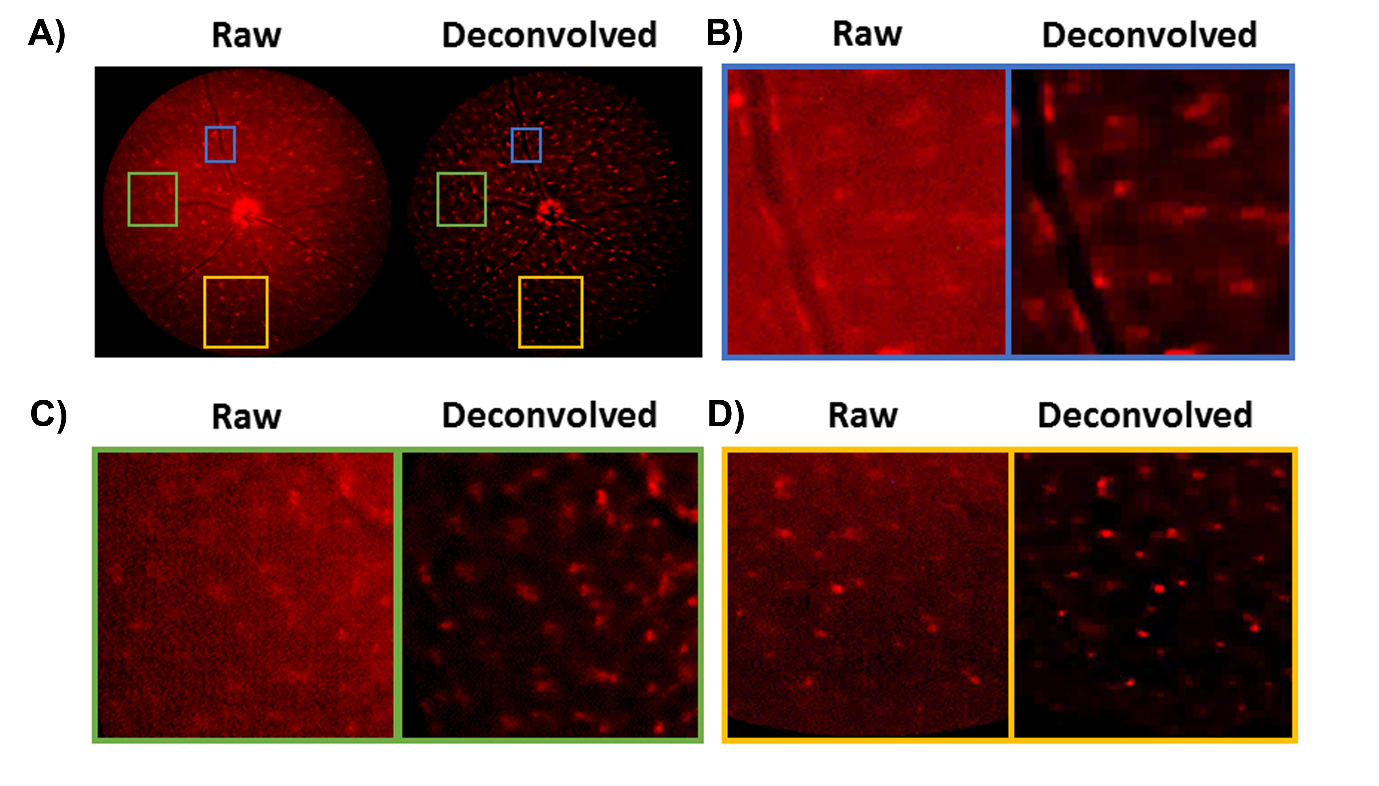


**Supplementary Figure 3. tdTomato fluorescence images of a mouse retina both pre- and post-deconvolution using the Huygens software. (A)** Fluorescent fundal images were acquired from naïve *Cx3cr1^CreER^:R26-tdTomato* mice 4 weeks following tamoxifen administration. Paired raw and deconvolved images showing how the software algorithm improves the image detail quality. Full fundal distribution of microglia and close-up images of cells located in **(B)** peri-vascular, **(C)** central and **(D)** peripheral retinal regions.


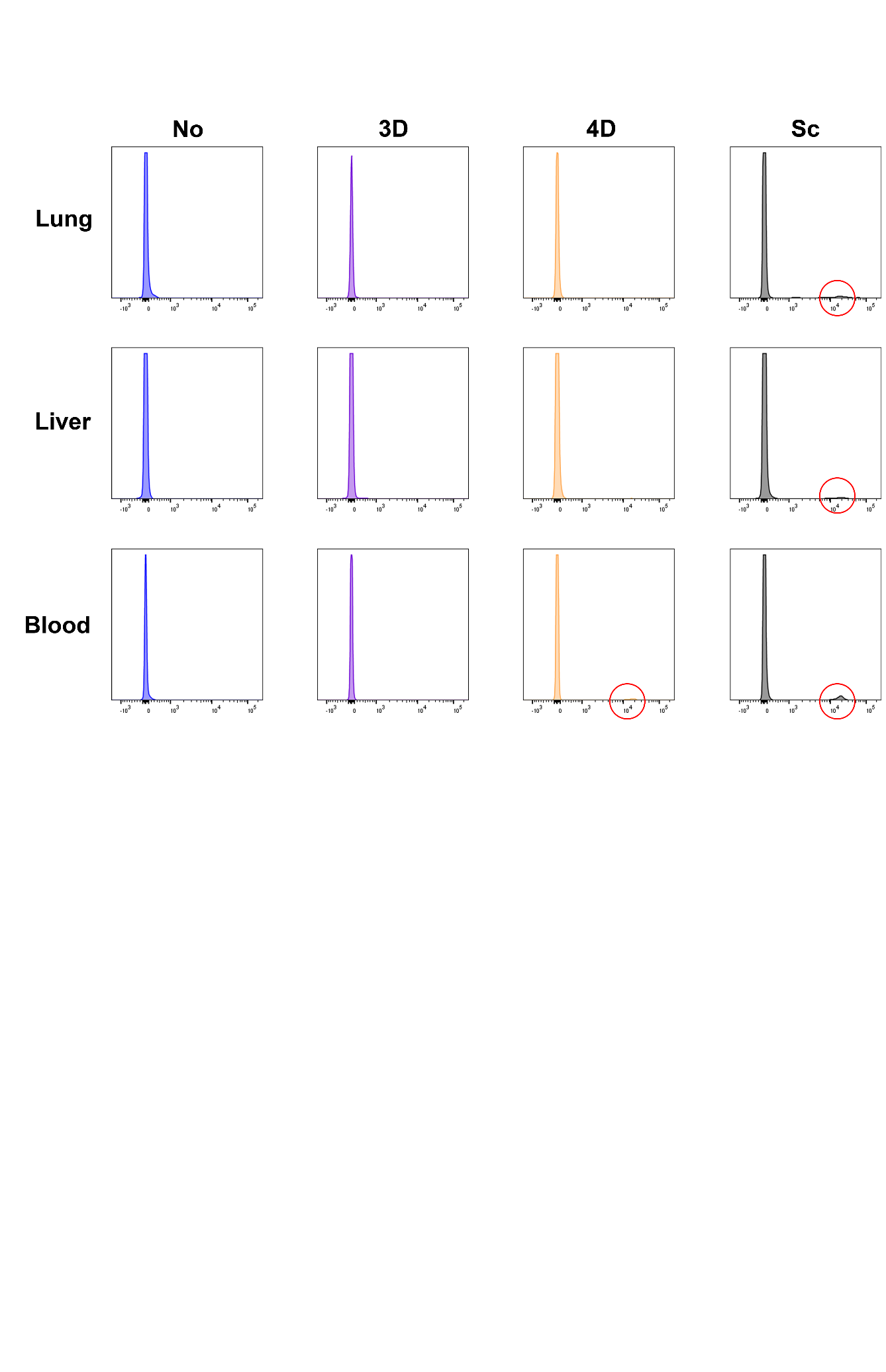


**Supplementary Figure 4. Flow cytometric analysis of peripheral tissues indicates a small, but potentially significant, number of tdTomato^hi^ cells.** Tissues were gated for cells, singlets, live cells, and then for myeloid cells (CD45^+^, CD11b^+^). Histograms of tdTomato fluorescence intensity from the different tissues are shown. Histograms containing a small number of positive cells are highlighted with circles. Abbreviations: No – no tamoxifen administered, 3D – 3-day topical tamoxifen regime, 4D – 4-day topical tamoxifen regime, Sc – subcutaneous tamoxifen regime.


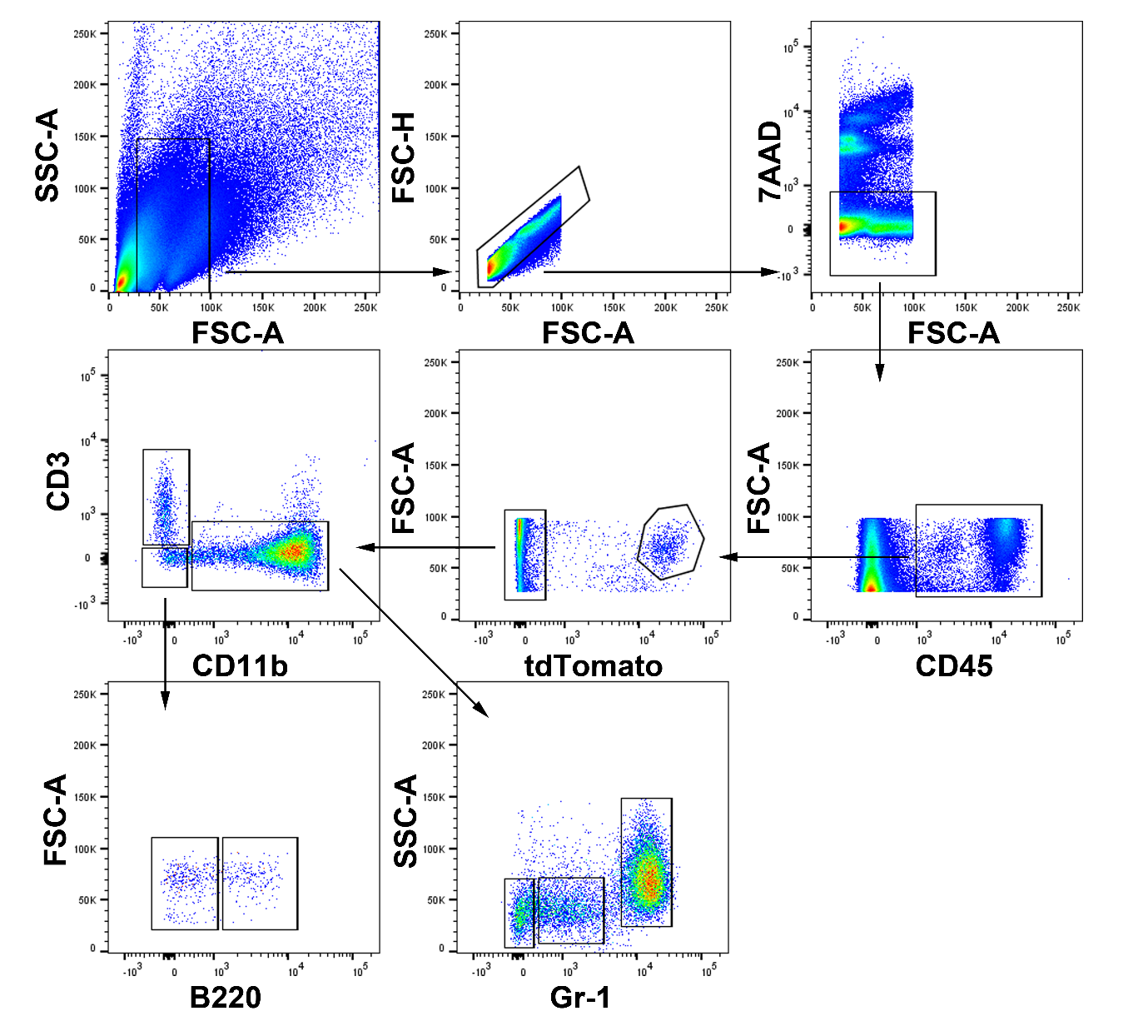


**Supplementary Figure 5. Flow Cytometric Gating Strategy to show differentiation of immune cell infiltrate and microglia at 18 hours EIU.** Retinas were gated for cells, singlets, live cells, and immune cells. They were then separated into microglia (tdTomato^hi^) and non-microglial cells (tdTomato^-^), myeloid cells (CD11b^+^), T cells (CD3^+^), and other (CD3^-^, CD11b^-^), with the myeloid cells further discriminated into Gr-1^-^, Gr-1^lo^ (Ly6C^+^), and Gr-1^hi^ (Ly6G^+^) cells. The “other” cells were gated on B220 to identify B cells.


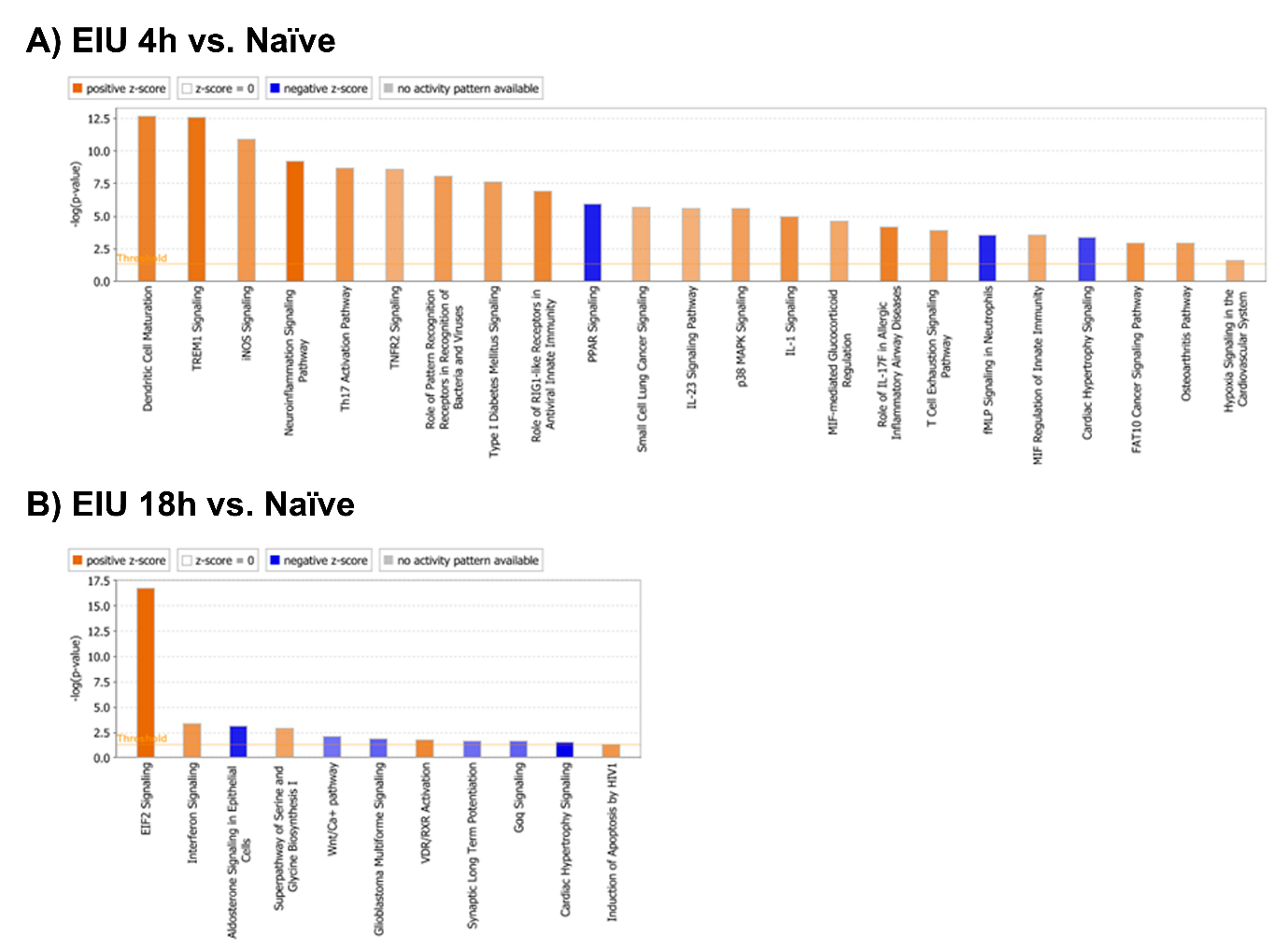


**Supplementary Figure 6. Ingenuity Pathway Analysis** (**IPA) software identifies canonical pathways that are significantly altered in microglia during the course of endotoxin-induced uveitis (EIU). (A)** Significantly altered pathways at 4 hours post-injection and **(B)** 18 hours post-injection are shown. A p value threshold of ≤0.05 is indicated by the horizontal line. The shading intensity of the bars indicates how strongly positive (orange) or negative (blue) the z-score (directionality score) was for each pathway.


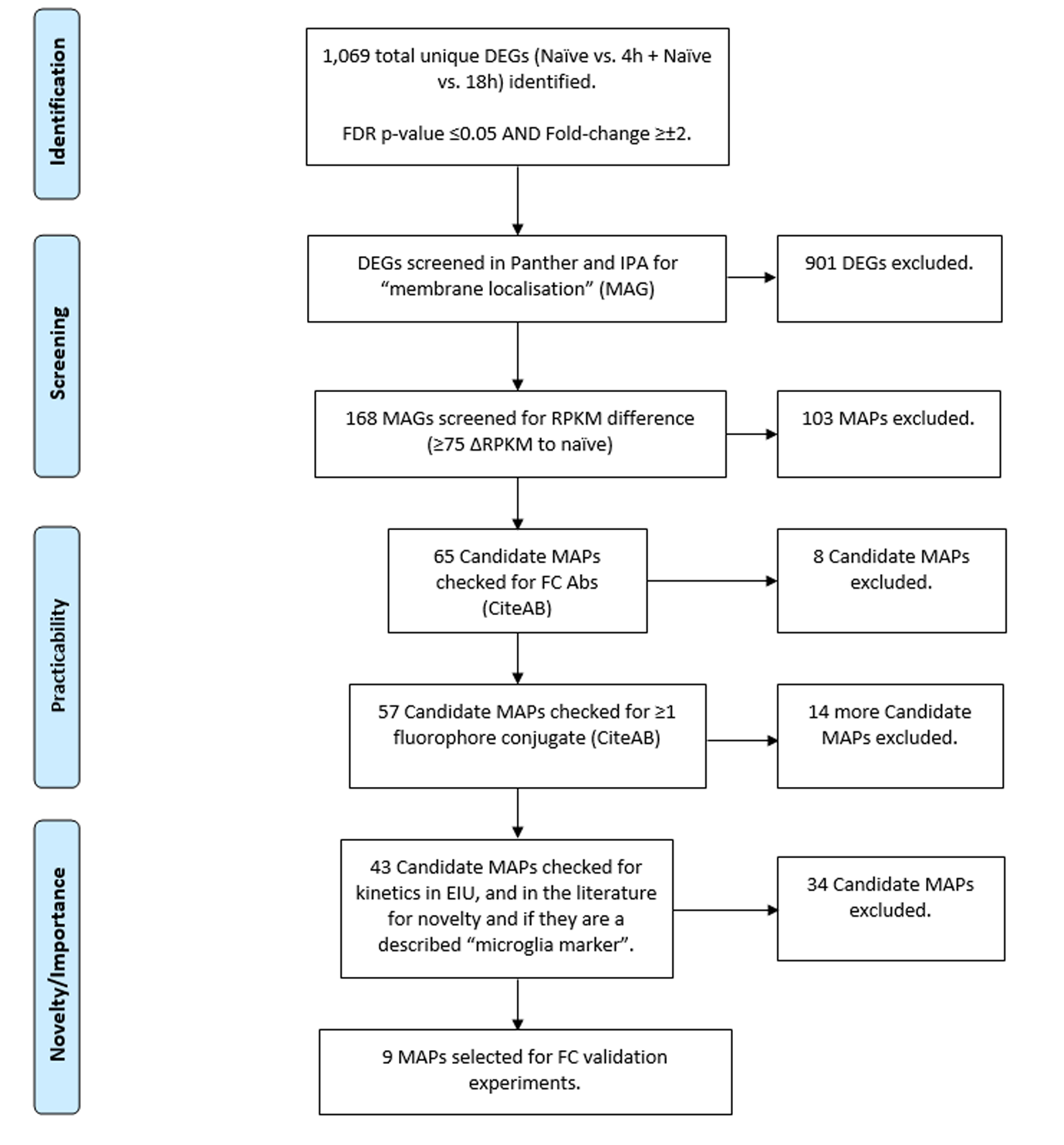


**Supplementary Figure 7. A flow-chart to demonstrate selection of markers for validation.** One thousand and sixty-nine DEGs identified and targets screened for membrane localisation (for simple flow cytometric validation), large RPKM difference, and availability of conjugated antibodies. Targets were selected based on whether they were novel with respect to describing microglial activation (*Milr1*) or without validation beyond the transcript level (*Bst2*, *Fas*, *Lair1*, *Slamf1*) (1-5), whether they were a previously specific or enriched microglial marker (*P2ry12*, *Siglech*) (6, 7), whether our data was in contrast to previous reports (*Mertk*) (8), or appeared crucial in light of other reports (*C5ar1*) (9). The other marker (*Cd44*) was selected for testing based on results of a pilot study and its previous description as a possible microglial marker (10). This diagram was generated using the PRISMA word document template (11). Abbreviations: DEG – differentially-expressed gene, MAG – membrane-associated gene, MAP – membrane-associated protein, FC – flow cytometry.

References:

1. Rangaraju S, Dammer EB, Raza SA, Rathakrishnan P, Xiao H, Gao T, et al. Identification and therapeutic modulation of a pro-inflammatory subset of disease-associated-microglia in Alzheimer's disease. Mol Neurodegener. 2018;13(1):24.

2. Vogt M, Bauer MK, Ferrari D, Schulze-Osthoff K. Oxidative stress and hypoxia/reoxygenation trigger CD95 (APO-1/Fas) ligand expression in microglial cells. FEBS Lett. 1998;429(1):67-72.

3. Das A, Chai JC, Kim SH, Park KS, Lee YS, Jung KH, et al. Dual RNA sequencing reveals the expression of unique transcriptomic signatures in lipopolysaccharide-induced BV-2 microglial cells. PLoS One. 2015;10(3):e0121117.

4. Izzy S, Liu Q, Fang Z, Lule S, Wu L, Chung JY, et al. Time-Dependent Changes in Microglia Transcriptional Networks Following Traumatic Brain Injury. Front Cell Neurosci. 2019;13:307.

5. Haslund-Vinding J, McBean G, Jaquet V, Vilhardt F. NADPH oxidases in oxidant production by microglia: activating receptors, pharmacology and association with disease. Br J Pharmacol. 2017;174(12):1733-49.

6. Butovsky O, Jedrychowski MP, Moore CS, Cialic R, Lanser AJ, Gabriely G, et al. Identification of a Unique TGF-β Dependent Molecular and Functional Signature in Microglia. Nature neuroscience. 2014;17(1):131-43.

7. Chiu IM, Morimoto ET, Goodarzi H, Liao JT, O'Keeffe S, Phatnani HP, et al. A neurodegeneration-specific gene-expression signature of acutely isolated microglia from an amyotrophic lateral sclerosis mouse model. Cell Rep. 2013;4(2):385-401.

8. Nomura K, Vilalta A, Allendorf DH, Hornik TC, Brown GC. Activated Microglia Desialylate and Phagocytose Cells via Neuraminidase, Galectin-3, and Mer Tyrosine Kinase. Journal of immunology (Baltimore, Md : 1950). 2017;198(12):4792-801.

9. Sousa C, Golebiewska A, Poovathingal SK, Kaoma T, Pires-Afonso Y, Martina S, et al. Single-cell transcriptomics reveals distinct inflammation-induced microglia signatures. EMBO Rep. 2018;19(11).

10. Lewis ND, Hill JD, Juchem KW, Stefanopoulos DE, Modis LK. RNA sequencing of microglia and monocyte-derived macrophages from mice with experimental autoimmune encephalomyelitis illustrates a changing phenotype with disease course. J Neuroimmunol. 2014;277(1-2):26-38.

11. Moher D, Liberati A, Tetzlaff J, Altman DG, Group P. Preferred reporting items for systematic reviews and meta-analyses: the PRISMA statement. PLoS Med. 2009;6(7):e1000097.
